# The Use of Proton Pump Inhibitors in Patients with Liver Cirrhosis: Real Life Experience

**DOI:** 10.3390/jcm13175155

**Published:** 2024-08-30

**Authors:** Raphaël Eftimie Spitz, Stefan Lucian Popa, Simona Grad, Dan Lucian Dumitrascu, Abdulrahman Ismaiel, Teodora Surdea-Blaga

**Affiliations:** 1 Faculty of Medicine, “Iuliu Hatieganu” University of Medicine and Pharmacy, 400000 Cluj-Napoca, Romania; raphael.spitz.e@gmail.com (R.E.S.); costinsimona_m@yahoo.com (S.G.); dan_dumitrascu@yahoo.de (D.L.D.); abdulrahman.ismaiel@yahoo.com (A.I.); dora_blaga@yahoo.com (T.S.-B.); 2 2nd Department of Internal Medicine, Emergency County Hospital, 400003 Cluj-Napoca, Romania

**Keywords:** proton pump inhibitors, liver cirrhosis, hepatotoxicity, drugs, liver injury, adverse drug reaction

## Abstract

(1) **Background**: Proton pump inhibitors (PPIs) are commonly prescribed for gastric disorders. In patients with liver cirrhosis, PPI use is associated with an increased risk of spontaneous bacterial peritonitis and increased mortality rates; therefore, they should be used with caution. This study aims to evaluate the appropriateness of PPI prescriptions in hospitalized cirrhotic patients against current clinical guidelines to identify patterns of misuse and guide better prescribing practices. (2) **Methods**: A retrospective study was conducted on liver cirrhosis inpatients in an internal medicine department from January 2022 to May 2023. The primary measure was the proportion of PPI prescriptions aligned with clinical guidelines. Medical files were entirely reviewed by researchers to assess the appropriateness of PPI prescriptions using the current guidelines. Outcomes included the identification of common reasons for PPI prescription and the rate of inappropriate PPI use among the study population. (3) **Results**: The study included 189 cirrhotic patients, with PPIs prescribed to 95 (50.2%) patients during hospitalization and 75 (39.7%) patients at discharge. Among those, 47.4% of the inpatients and 34.7% at discharge had no valid indication for PPI administration. The most common reason for PPI prescription during hospital stays was gastritis, followed by antiplatelet use in high-risk patients, ulcers, and upper gastrointestinal bleeding. The most common inappropriate indication was portal hypertensive gastropathy (PHG), followed by treatment with corticosteroids and anticoagulants alone. We did not find an association between PPI administration during hospital stays and infections. Only in 4% of cases patients should have received PPIs and did not. (4) **Conclusions**: There is a concerning overprescription of PPIs in cirrhotic patients, often deviating from established guidelines. It subjects patients to unnecessary risks. There is an urgent need for increased awareness and adherence to clinical guidelines regarding PPI prescriptions in cirrhotic patients.

## 1. Introduction

Liver cirrhosis can result from various factors, including alcohol consumption, chronic infection with hepatitis viruses, primary biliary cholangitis, autoimmune disorders, or when the origin is cryptogenic. It can lead to numerous complications, like upper gastrointestinal bleeding (UGIB), ascites, and hepatocellular carcinoma, and patients are at an increased risk of mortality [1,2]. Several patient-related factors, such as alcohol use, advanced age, type 2 diabetes, or being overweight, contribute to increased morbidity and mortality [3]. Another host-related factor is the disturbance in the composition of the gut microbiota and dysbiosis, which is involved in the pathogenesis of hepatic encephalopathy and spontaneous bacterial peritonitis (SBP) [4].

PPIs have been in use for over 30 years and are widely perceived as a safe option by many healthcare providers [5]. PPIs reduce stomach acid secretion, thereby mitigating the associated risks. Nonetheless, there are drawbacks to utilizing PPIs: they can alter the gut microbiota, leading to dysbiosis, which, in turn, triggers intestinal inflammation and enhances intestinal permeability [6]. Prolonged use of PPIs increases the risk of community-acquired pneumonia, especially during the first month of therapy, and the risk of hospitalization for pneumonia (OR 1.61; 95% CI: 1.12–2.31), as shown by published metanalyses [7]. In patients with liver cirrhosis, some studies reported an increased risk of SBP in patients who took PPIs within 7 days before hospitalization [8]. A recent meta-analysis reported an RR of 1.31 (95% CI, 1.10–1.55) for SBP and 1.37 (95% CI 1.07–1.76) for overall infection risk in cirrhotic patients who use PPIs [9]. PPI exposure of 1 month increases the 1-month and 3-month hospital readmission rate and hepatic decompensation of liver cirrhosis (aRR = 1.61, (1.30–2.11); *p* < 0.001). Longer PPI exposure (>90 days) was associated with increased mortality (HR = 2.10, (1.20–3.67); *p* = 0.009) [10]. Even in short-term use, studies suggest acid rebound, defined as increased gastric acid production surpassing pre-PPI treatment levels [11]. There is an increasing trend in PPI prescriptions in internal medicine and gastroenterology [12]. In patients with liver cirrhosis, 63.6% had been prescribed PPIs, and among these, 62.7% had no clear indication for the prescription [13]. 

On the one hand, there is widespread endorsement of this medication by numerous healthcare professionals over time; on the other hand, concerns about its potential complications have been raised. These recent studies underscore the critical balance between the advantages and risks, highlighting the necessity of prescribing PPIs with precise indications, particularly in patients with liver cirrhosis. The objective of this study was to ascertain the frequency of PPI prescriptions in hospitalized patients with liver cirrhosis and establish the appropriateness of the indications and the route of PPI administration. The secondary objectives were 1. to analyze the various indications for PPI prescription at discharge and the duration of the prescription; 2. for patients without PPIs, we examined whether the treatment for their associated conditions could have required PPI administration but was omitted by physicians.

## 2. Materials and Methods

### 2.1. Study Population 

This study was retrospective and included consecutive liver cirrhosis inpatients admitted to the 2nd Medical Department of our hospital between January 2022 and 15 May 2023. The following ICD codes were used for the selection of the patients: K70.0 “Alcoholic fatty liver cirrhosis”, K70.3 “Alcoholic liver cirrhosis”, K71.7 “Toxic liver disease with fibrosis and cirrhosis of the liver”, K74.0 “Hepatic fibrosis”, K74.1 “Hepatic sclerosis”, K74.2 “Hepatic fibrosis with hepatic sclerosis”, K74.3 “Primary biliary cirrhosis”, K74.4 “Secondary biliary cirrhosis”, K74.5 “Liver cirrhosis, unspecified”, and K74.6 “Other and unspecified cirrhosis of the liver”. The healthcare team responsible for treating hospitalized patients comprised five gastroenterologists and twelve internal medicine physicians.

### 2.2. Definitions

For every patient, we recorded the type of PPI prescription: PPI treatment during their hospitalization (patients who received at least one day of PPI treatment during their hospitalization) and PPI at discharge (patients who were discharged with a prescription for a PPI).

To evaluate the appropriateness of these prescriptions, we employed the consensus on PPIs published by Scarpignato et al. [14] in 2016. This consensus discusses both PPI indications for treatment (in GERD, PUD, etc.) and prophylaxis and includes all topics from various individual guidelines, such as those from NICE [15] or the American College of Gastroenterology guidelines [16,17] that are used in other studies [13,18,19,20].

We considered an indication to be valid when the PPI was administered for one of the following: the treatment of gastro-esophageal reflux disease (GERD) (with erosive esophagitis or PPI responsive non-erosive GERD), UGIB, Non-Steroid-Anti-Inflammatory Drug (NSAID)-induced ulcers, PUD, gastritis or duodenitis, HP eradication, functional dyspepsia, prevention of NSAID-induced ulcers in patients with previous history of UGIB, and Stress Ulcer Prophylaxis (SUP) in high-risk patients (critically ill patients with respiratory failure or coagulopathy). In addition, the administration of PPIs in patients using antiplatelet therapy and with a high risk for upper GI complications was valid in the following cases: age > 65 years, patients taking two antiplatelet drugs, concurrent usage of NSAIDs and/or steroids, concomitant use of an anticoagulant, or a history of prior UGIB. Concomitant NSAID and corticosteroid treatment was also considered a valid indication based on the above-mentioned guidelines [14].

Each patient’s file was reviewed independently by two of the researchers to determine whether the prescription of PPIs was aligned with any of these criteria or not. We also considered no valid indication for the following two scenarios: 1. the indication was correct, but the duration of prescription was incorrect (either not mentioned by the physician or longer than the maximum duration reported in the consensus) [14]; or 2. there was an improper route of administration (intravenous when oral administration would have been sufficient). For the patients with no valid indication, we tried to identify a possible reason for prescribing a PPI, and the following categories were recorded: (i) “anticoagulant alone” for patients who had an anticoagulant but no antiplatelet was associated, (ii) “antiplatelet low risk” for patients who did not fit in any high-risk category, as mentioned above; (iii) “NSAID alone” for those who had NSAID without prescription for corticosteroids or antiplatelets, (iv) “corticosteroid alone” for those who had corticosteroid without prescription for NSAID or antiplatelets, and (v) “too long duration of prescription” for a correct indication but the duration for prescription surpassed the duration recommended by the guidelines [14]. The remaining cases were included in the category (vi) “no indication found”. When the same patient had been hospitalized multiple times, we considered each hospitalization separately. 

### 2.3. Data Collection and Assessment

All medical files were reviewed, and for each one, we collected age, gender, etiology of liver cirrhosis, date of admission, reason for hospitalization, relevant comorbidities (atrial fibrillation, pulmonary embolism, deep vein thrombosis, coronary artery disease, osteoarthritis, pneumonia, urinary tract infection, skin infection, GERD, and colitis with diarrhea), and complications of cirrhosis (hepatic encephalopathy, peritonitis, renal failure, hepatocellular carcinoma, esophageal varices, and UGIB). During hospitalization, we recorded the PPI prescription, the dose, and the indication. At discharge, we recorded the PPI prescription, the dose, the number of doses per day, the duration of the prescription, and the indication. 

### 2.4. Statistical Analysis 

Data are presented as mean ± standard deviation or percentage (%). For categorical variables, frequency tables were created, and *p*-values were calculated using the chi-squared test. The study protocol was approved by the Committee of Ethics of the University of Medicine and Pharmacy, Cluj-Napoca, Romania (No. 44 from 12 March 2024).

## 3. Results

### 3.1. Selection of Patients

**Figure 1:**
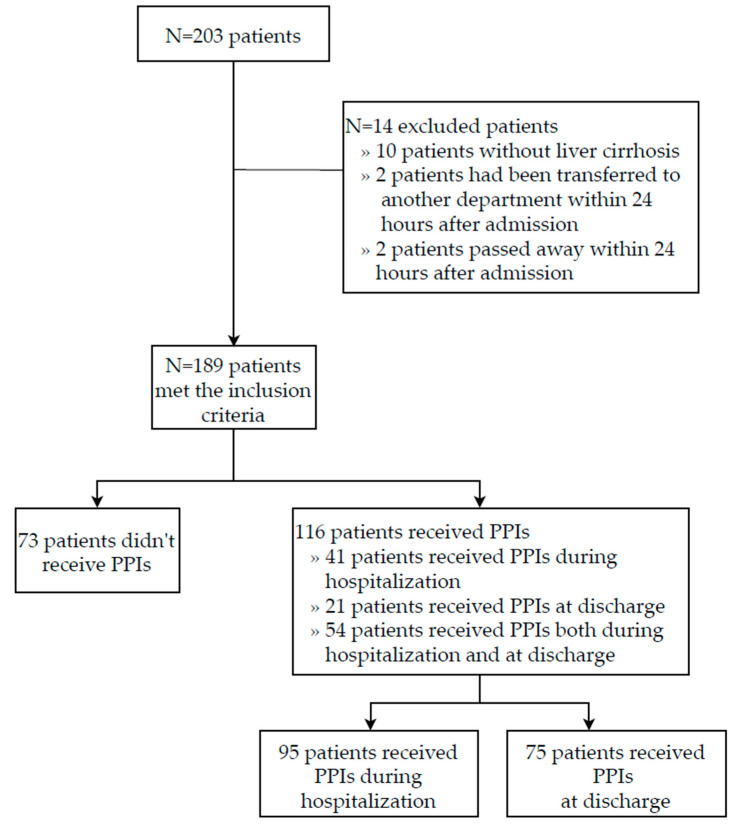
Selection of patients with liver cirrhosis and PPI prescriptions during hospital stay and at discharge.

The search retrieved a total of 203 patients. As shown in Figure 1, patients who met any of the following criteria were excluded: (i) patients who passed away within the first 7 days after admission; (ii) patients with other hepatic disease without liver cirrhosis; and (iii) patients transferred to another service within 24 h after admission. Based on these criteria, a total of 14 patients were excluded from the study. Of these, 10 patients had primary biliary cholangitis (but with no signs of cirrhosis), 2 were transferred to another department within 24 h after admission, and 2 passed away within 24 h after admission. Finally, a total of 189 consecutive patients were included in the current study. Among them, 113 patients received PPIs. We created two groups: 95 patients received PPIs during hospitalization, and 75 patients received PPIs at discharge. Ultimately, 54 patients received PPIs during hospitalization and at discharge.

### 3.2. Baseline Characteristics of Liver Cirrhosis Cohort 

The average age of patients was 63.1 ± 9.7 years, and most of the patients were males. Chronic alcohol use was the most frequent etiology for liver cirrhosis, accounting for 99 patients (52.4%). The other etiologies are presented in Table 1. Cirrhosis was compensated in 79 (41.8%) patients and severely decompensated in 31 (16.4%) patients.

### 3.3. Comorbidities in Liver Cirrhosis Cohort

**Figure 2:**
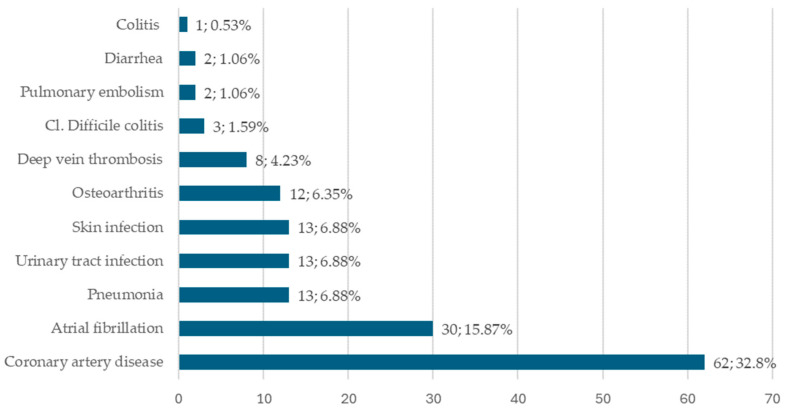
The prevalence of comorbidities reported in the cohort of cirrhotic patients (*n* = 189).

The most common comorbidity in our cohort was coronary artery disease, followed by infections and atrial fibrillation (Figure 2). The infections observed were pneumonia, urinary tract infections, and skin infections. In total, 43 patients (22.7%) had infections (including SBP). Three patients had Clostridioides difficile infection, while in two other patients, no etiologic factor was identified for diarrhea. One patient had non-infectious colitis (possible ischemic colitis or Crohn’s disease). Among the 94 patients on PPIs during hospital stays, 23 (24.4%) had infections, while 20 (27.4%) patients without PPIs (from the total of 73 patients) were diagnosed with an infection. The chi-square test showed no difference between the two groups (*p* = 0.63) regarding the frequency of infection in relation to PPIs.

The most frequent complication of cirrhosis was esophageal varices, present in 104 (55.0%) patients, followed by ascites, hepatic encephalopathy, and renal failure. Other complications, like UGIB, hepatocellular carcinoma, or peritonitis, were present in less than 5% of cases (Table 2).

### 3.4. PPI Prescription during Hospital Stays and at Discharge and the Validity of Prescription

**Figure 3:**
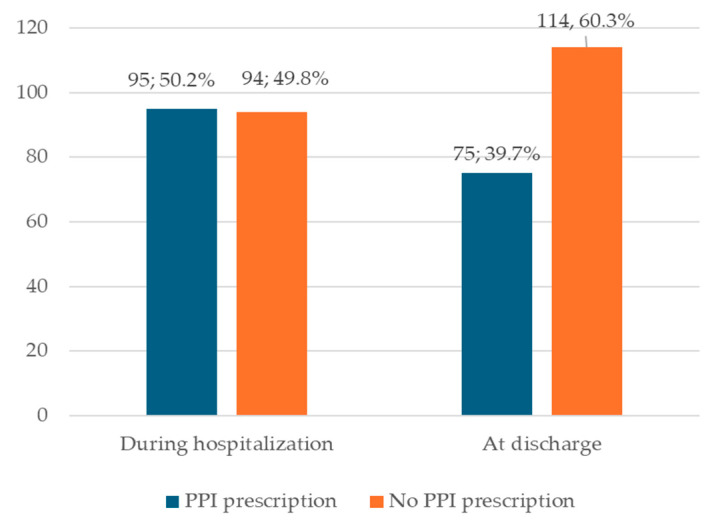
The number of patients with liver cirrhosis who were prescribed PPIs during hospitalization and at discharge.

During hospitalization, 95 (50.2%) inpatients received PPIs, whereas 75 (39.7%) received it at discharge (Figure 3).

**Figure 4:**
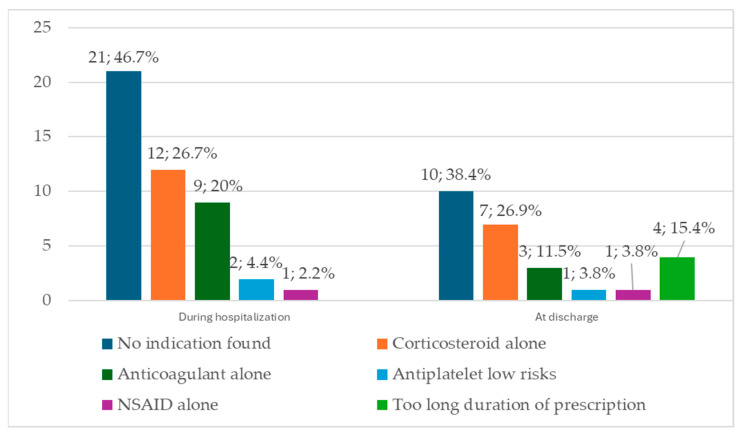
Reasons for non-valid indication for PPI use. During hospital stays, 45 patients did not have a valid indication, and results are presented as absolute and relative frequency (out of 45 patients). At discharge, there were 26 patients without a valid indication for PPI use; results are presented as absolute and relative frequency (out of 26 patients).

During hospitalization, 45 of the 95 (47.4%) patients on PPIs did not have a valid indication for their use. Among these, 21 patients (46.7%) had no identified reason for PPI use documented in their medical file. For the remaining 24 patients, the reasons for considering the indications non-valid are presented in Figure 4. Twelve patients received corticosteroids during the hospital stay, nine had anticoagulants, two received antiplatelet therapy, and one patient received NSAID alone. Among the 21 patients with no indication found for PPI prescription, 14 patients had portal hypertensive gastropathy (PHG).

At discharge, of the 75 patients who received PPIs, 26 (34.7%) patients did not have a valid indication for their use, including 10 (38.4%) with no indication at all. Five out of ten patients with no indication found had PHG. Figure 4 depicts the reasons for considering the indications for PPI prescription “non valid” at discharge.

### 3.5. The Appropriate Indications for PPI Prescription in the Cohort of Patients with Liver Cirrhosis 

**Figure 5:**
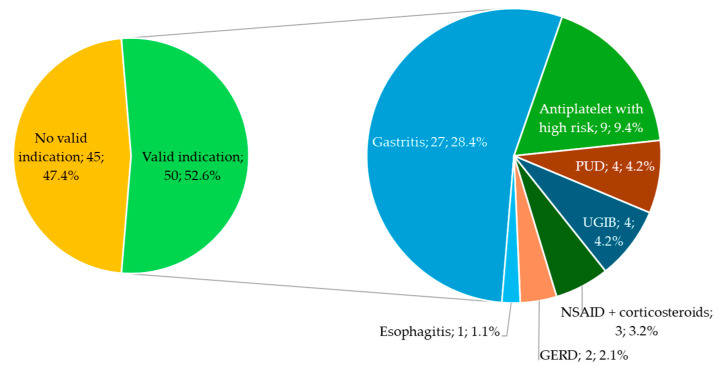
Indications for PPI prescription during hospitalization. The relative frequencies are out of a total of 95 patients who received PPIs during hospital stays; PUD, peptic ulcer disease; UGIB, upper gastrointestinal bleeding; NSAID, non-steroid anti-inflammatory drugs; GERD, gastroesophageal reflux disease.

**Figure 6:**
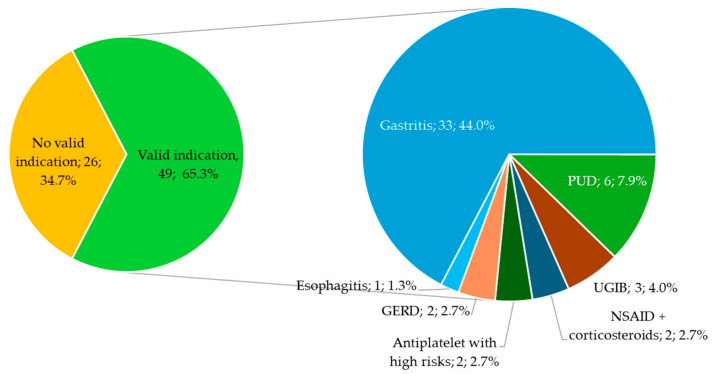
Indications for PPI prescription at discharge. The relative frequencies are out of a total of 75 patients who received PPIs at discharge. PUD, peptic ulcer disease; UGIB, upper gastrointestinal bleeding; NSAID, non-steroid anti-inflammatory drugs; GERD, gastroesophageal reflux disease.

The most common indication for PPI prescription was gastritis. This was reported in 27 (28.4%) patients during their hospital stays and in 33 (44.0%) patients at discharge. The second most common indication for PPI prescription during hospital stays was the use of antiplatelet drugs in patients with a high risk of GI complications, comprising 11 patients. The other valid indications for PPI prescription are presented in Figure 5 and Figure 6.

### 3.6. Patients without PPI Treatment during Hospital Stays or at Discharge

Among the 73 patients with liver cirrhosis who did not receive PPIs during hospital stays or at discharge, 3 (4.1%) patients should have received PPIs for the following reasons: concomitant NSAID and corticosteroids administration (n = 1) and concomitant antithrombotic + anticoagulant treatment (n = 2).

### 3.7. Route of Administration

Of the 95 patients who received PPIs during hospital stays, only 2 patients were prescribed PPIs by intravenous route. This was justified in their files by difficulties in feeding due to swallowing disturbance. The remaining patients received oral PPIs. 

## 4. Discussion

Our paper presents three major findings. The first major finding is that almost half of the patients had no valid indication for PPI administration during hospitalization and about one-third had no valid indication at discharge. The second major finding reveals that, for inpatients on PPIs, the most common invalid indication was prophylaxis against portal hypertensive gastropathy (PHG), followed by the use of corticosteroids alone, and use of anticoagulants alone. PHG was the second reason for no valid indication at discharge, after corticoid alone. The third major finding indicates that, among all patients who took PPIs, the most frequently cited reason for prescription was the presence of gastritis.

Concerning the valid indications for PPIs, the results are consistent with the findings of other studies carried out elsewhere [13,14,15,16,17,18,19,20,21]. A European study identified an inappropriate PPI usage rate of 61.5%, with “anticoagulant alone” as the leading unjustified reason, a finding that aligns with our study [18]. Similarly, research in Singapore showed a 54.1% inappropriate PPI use rate, but adherence to NICE and ACG guidelines reduced this figure slightly [18]. A study that addressed particularly PPI use in liver cirrhosis [13] included patients from a hepatology ward. They assessed the discharge forms and a list of patients assessed for orthotopic liver transplant (OLT). The research reported that more than 60% of patients had PPIs prescribed at discharge, with 62.7% of patients without a clinical diagnosis in accordance with the NICE guidelines for PPI prescription. Similarly, among patients listed for OLT, 73.7% had no indication for PPI prescription. The paper did not provide data on comorbidities that might necessitate chronic treatment with drugs that increase the risk of hemorrhage. Only patients taking PPIs for acid-related gastrointestinal conditions (gastritis, duodenitis, ulcer, GERD, and Barrett’s esophagus) were listed by authors as valid indications, and the NICE guidelines used as a reference cover only these aspects. The cohort in that study had a younger average age (56 years) compared to ours, suggesting a potentially lower prevalence of cardiovascular comorbidities. The authors believed that PPIs were prescribed to relieve abdominal discomfort, which is common in cirrhotic patients [13]. Similarly, Dultsz et al. reported that in their cohort of cirrhosis patients, 78.3% of patients were on PPIs, and 58% had PPIs for epigastric pain or abdominal discomfort. The authors also reported that PPI treatment is associated with higher mortality [20]. Once again, the other valid indications (i.e., concomitant antiplatelet and anticoagulant therapy) [13] were not assessed. These studies collectively highlight a global trend of PPIs being frequently prescribed without valid indications.

PPIs are shown to decrease the risk of UGIB among users of low-dose aspirin for cardiovascular disease prophylaxis [22]. According to the current guidelines [14], not all patients taking an antiplatelet agent are recommended to receive a PPI; it is specifically indicated for those considered at a high risk of bleeding due to their age or the association of other drugs that increase the risk of GI bleeding (like NSAIDs, corticosteroids, or anticoagulants). The presence of liver cirrhosis or PHG is not typically considered a risk factor. Only a few of our patients (two during their hospital stay and one at discharge) were likely prescribed a PPI primarily due to their chronic antiplatelet therapy, as we were unable to identify any other risk factors. However, this number is relatively small in comparison to the number of patients for whom we could not discern any objective apparent reason for PPI administration, except for the presence of PHG. Our study lacked data on the specific symptoms of patients, which other studies have identified as a contributing factor to PPI overprescription [13,20]. Most patients without a clear indication for PPI prescription during their hospital stay had PHG identified during upper GI endoscopy, leading us to believe that this might have been the rationale behind the PPI prescription. Similarly, at discharge, PHG was the second reason for no valid indication. According to the consensus of Scarpignato [14], there is no evidence that PPIs prevent UGIB bleeding due to PHG, as it is not acid-related; therefore, PPIs should not be routinely prescribed in this condition [22,23]. There are few studies on this subject, and one of them [24] concluded that in cirrhotic patients, PPIs do not reduce the bleeding risk associated with portal hypertension. Gastric acid secretion is reduced in patients with liver cirrhosis, gastric acid secretion is already reduced [25,26], and further suppression with PPIs can lead to changes in microbiota and bacterial overgrowth [27].

In liver cirrhosis, a delicate balance between coagulation and hemorrhage exists [28,29]. Patients with liver cirrhosis may face risks of both hemorrhage and thrombosis, yet, as of now, accurate laboratory tests for assessing these risks are lacking. Physicians often exhibit reluctance to prescribe anticoagulants in liver cirrhosis due to the associated risk of life-threatening bleeding [30]. Bleeding is the most prevalent adverse effect of anticoagulants [31]. While anticoagulants do not elevate the risk of variceal bleeding in patients with cirrhosis [32], they can induce nonvariceal GI bleeding or bleeding at other sites (retroperitoneal, intracranial, nasal, urinary, etc.) like in any other patient. Despite these risks, cirrhotic patients should receive anticoagulants, if necessary, like any other patients. Concomitant PPI treatment during anticoagulant therapy to prevent UGIB is not currently recommended [14,15]. Nevertheless, several studies report that PPIs reduced UGIB episodes in patients with chronic anticoagulant treatments [33], particularly when patients had a prior history of gastrointestinal bleeding [34]. In our study, one of the reasons for overprescribing PPIs was chronic treatment with an anticoagulant in the presence of liver cirrhosis. Most probably, clinicians adopted this measure as an extra precaution to prevent GI bleeding episodes, although to date, the data are insufficient to support this approach. According to the current guidelines [14,15,16,17], it is not advisable to routinely suggest the use of PPIs for individuals who are on anticoagulant medication. 

Another non-valid indication for PPIs in our study was treatment with “corticosteroids alone” in the absence of other drugs that increase the risk of GI bleeding. The available evidence on the likelihood of gastrointestinal bleeding in patients taking corticosteroids is inconsistent [35,36,37]. Critically ill patients who are prescribed corticosteroids face an elevated risk of experiencing “clinically significant” GI bleeding [35], but it is important not to generalize this observation to all patient populations. Once more, the existing guidelines do not advocate the routine use of PPIs in patients who are taking corticosteroids [14,15,16,17].

Regarding the under-prescription of PPIs, to our knowledge, there are few studies on the subject. A study from China [38] found that 9.3% (n = 112) of inpatients who required PPIs for Stress Ulcer Prophylaxis did not receive them. In our research, we found that only 4% of patients who were not using PPIs needed them. Interestingly, none of the previously mentioned studies [13,14,15,16,17,18,19,20] took this aspect into account when discussing the appropriateness of PPI utilization.

Our research reveals that nearly half of the patients with liver cirrhosis admitted to our department should not have been prescribed PPIs. It is important to note that our study focused on a particularly unique patient population consisting of individuals with liver cirrhosis. Furthermore, our cohort had a mean age of 63 years, with a significant proportion of patients also having coronary artery disease or atrial fibrillation. Portal hypertensive gastropathy by itself may not necessitate PPI treatment, although additional research may be needed to determine if this remains valid when coupled with antiplatelet or anticoagulant therapy.

PPIs increase the risk of infections [8,9,39]. One of the largest studies on this subject [39] considered both the daily dose and the total dose per month of each PPI class and reported that the risk of severe infection is higher with higher doses of PPIs. PPIs increase not only the risk of infection but also the risk of decompensation and, therefore, might contribute to a higher risk of liver-related death [39]. However, when prescribed according to appropriate indications, PPIs have a protective effect in terms of survival. PPIs should be prescribed only when clinically indicated and at the lowest effective dose to achieve the desired therapeutic outcome [40,41]. We did not find an association between PPI administration during hospital stays and infections. However, an important proportion of our patients were diagnosed with a concomitant infection that may have contributed to the development of decompensated liver cirrhosis. During hospital stays, we identified three (1.6%) patients with Clostridioides difficile colitis, and two of them received PPIs, concomitant with antibiotic treatment. Additionally, we had one patient with neutrocitic ascites, and he had a PPI prescription during hospitalization and at discharge. 

Our data now demonstrate that despite existing concerns about the use of PPIs in individuals with cirrhosis, these drugs are still being widely prescribed without strict adherence to the established guidelines. It is true that except for the Italian consensus [14], the other existing guidelines [15,16,17] each cover a very specific topic regarding PPI use. Our study has several limitations derived from the retrospective design. We did not have access to information regarding the dose and the duration of prescriptions for PPIs during hospital stays. These data were not included in the electronic data that we used for this study. Extensive manual work would have been required for this specific information. We lacked information regarding the use of PPIs prior to hospital admission, making it difficult to make assumptions about any potential elevated infection risk associated with PPIs. Another limitation arises from the relatively small number of patients enrolled in this study and the fact that the research was exclusively conducted within one department of our hospital. Consequently, we cannot extrapolate the findings to apply universally across all healthcare settings. A further limitation is that we did not have data regarding the complications and outcomes of our patients possibly related to PPI use. We cannot follow up with patients, given the fact that we do not have a national medical electronic database, and our patients are free to consult any other hospital in the city or county. The absence of data on local prescribing patterns is a limitation of our study. Indeed, there are no specific national or local guidelines available for prescribing PPIs, especially for patients with liver cirrhosis. PPI overprescription among cirrhotic patients within our institution raises concerns about the potential adverse effects and unnecessary healthcare costs. Our institution should address the overuse of PPIs through several initiatives, like revising clinical guidelines, implementing educational programs for healthcare providers, and establishing a monitoring system to ensure adherence to best practices. These measures are intended to optimize PPI prescribing and enhance patient safety.

## 5. Conclusions

Our study revisits the critical question of PPI prescription practices for cirrhosis patients. It reveals that half of our patients with liver cirrhosis received PPIs during their hospital stay, but more than 40% of PPI prescriptions, either during hospitalization or at discharge, lacked valid indications based on the current guidelines. This aligns with other studies indicating inappropriate PPI usage in similar patient populations. The most frequent invalid indication for PPI prescription was prophylaxis against PHG, both during hospitalization and at discharge. This practice persists despite the lack of evidence supporting PPI effectiveness in preventing UGIB due to PHG. Other frequent invalid indications included the use of corticosteroids or anticoagulants alone, although the guidelines recommend PPI use alongside these medications only for patients at a high risk of GI bleeding due to other risk factors. Our data indicate that there is a PPI overprescription, diverging from the guidelines. Very rarely, there was an under-prescription of PPIs. The most common valid indication for prescribing PPIs was gastritis. While appropriate, this finding highlights the need to differentiate between valid and potentially unnecessary PPI prescriptions. Our study underscores the urgent need to increase awareness among healthcare providers regarding appropriate PPI prescribing practices for patients with liver cirrhosis. This includes strictly adhering to the established guidelines and considering the potential risks associated with PPI use in this patient population, especially regarding increased infection risk. By addressing the knowledge gap identified in this and similar studies, healthcare providers can ensure that PPI prescriptions are reserved for situations where the potential benefits outweigh the risks.

## Figures and Tables

**Table 1:** Baseline patient characteristics.

Variable		Liver Cirrhosis Patients (N = 189)
Age, Mean ± SD [Min–Max]		63.1 ± 9.7 (41–89)
Gender, No. (%)		
	Male	127 (67.2)
	Female	62 (32.8)
Cirrhosis etiology, No. (%)		
	Alcohol	99 (52.4)
	Hepatitis C	32 (16.9)
	Combined causes	16 (8.5)
	Cryptogenic	14 (7.4)
	Autoimmune	12 (6.4)
	Primary biliary cholangitis	9 (4.8)
	Hepatitis B	6 (3.2)
	Drug-induced	1 (0.5)
Child–Pugh Class, No. (%)		
	A	79 (41.8)
	B	71 (37.6)
	C	31 (16.4)
	Unknown	8 (4.2)

**Table 2:** Complications of liver cirrhosis in the study group (n = 189).

Complications of Liver Cirrhosis				No. (%)
	Ascites			
		Absent/mild		136 (72)
		Moderate		22 (11.6)
		Severe		31 (16.4)
	Hepatic encephalopathy			
		Absent		158 (82.5)
		Mild/moderate (Stage I–II)		26 (14.8)
		Severe (Stage III–IV)		5 (2.7)
	Esophageal varices			
		Yes		104 (55)
			Grade I	36 (19.1)
			Grade II	57 (30.1)
			Grade III	8 (4.2)
			Unknown	3 (1.6)
		No		27 (14.3)
		Gastroscopy not performed		58 (30.7)
	Renal failure			29 (15.3)
	Hepatocellular carcinoma			
		No		177 (93.6)
		Yes		6 (3.2)
		Possible (uncharacterized nodule)		6 (3.2)
	Upper gastrointestinal bleeding			9 (4.8)
	Spontaneous bacterial peritonitis			1 (0.5)

## Data Availability

Further information can be obtained by contacting the corresponding author.
